# The Ethics of Innovation: Ethical Decision-Making and Review for Field Studies and Projects Targeting Dogs and Cats

**DOI:** 10.3390/ani11123579

**Published:** 2021-12-17

**Authors:** Valerie A. Benka

**Affiliations:** Alliance for Contraception in Cats and Dogs, 2815 NE 66th Avenue, Portland, OR 97213, USA; valerie@acc-d.org

**Keywords:** animal ethics, animal research, animal welfare, companion animals, domestic cat, domestic dog, ethical decision-making, ethical review, ethics

## Abstract

**Simple Summary:**

Innovation and research to advance animal welfare, particularly that of companion animal species, can present unique ethical challenges, and there are presently gaps in support and independent oversight structures for ethical decision-making. This commentary details key gaps and one organization’s creation of an Ethical Review Board and structure for ethical review to help address them.

**Abstract:**

To date, independent ethical oversight of many companion animal welfare initiatives has been limited and, in some instances, inadequate. Beyond a blurred line between “innovation” and “research,” the nature of the work conducted in animal welfare projects is often poorly aligned with established institutional ethical review structures, which are designed for research involving humans or research involving animals and are also focused on industry and academic institutions. This commentary details the struggle of one United States-based nonprofit organization to find ethical guidelines and support for conducting non-traditional field-based animal welfare studies, and subsequent experience establishing an Ethical Review Board to evaluate organizational initiatives. The commentary discusses member selection, materials and processes, and lessons and learnings from the creation and use of an Ethical Review Board. Sharing content of the ethical review process, as well as challenges and learnings from it, is intended to support other organizations and individuals seeking to ensure that innovation for animal welfare consistently meets high ethical standards.

## 1. Introduction

Dog and cat welfare emphasizes innovation to help animals, and indeed significant funding is directed toward this focus (see, e.g., [1,2,3]). Innovation is both valuable and essential to advancing animal welfare. However, like anything novel, innovation carries some degree of risk to participants, and this in turn prompts ethical considerations and concerns.

To date, independent ethical oversight of companion animal welfare initiatives in the United States and other countries has been limited and, in some instances, inadequate [4]. A variety of factors contribute to this, beginning with an often-blurred line in animal welfare between “innovation” and “research.” While the projects that are a focus of the following commentary would reasonably be called “research,” many projects conducted by organizations focused on the welfare of companion animal species would not. Examples include initiatives to increase sterilization and rabies vaccination numbers, to better engage pet guardians and improve access to veterinary care, to reinvent outdated animal sheltering practices, to increase adoptions and otherwise enhance lifesaving, or to develop new products for companion animal species. They would simply be called “innovation” or “advancement.” Yet much of what is being done in these aforementioned projects is effectively research insofar as the experiences and outcomes for target populations are unknown. This necessitates reflection on how the animal welfare community can best protect the vulnerable populations that it seeks to benefit through new projects and novel ideas.

In addition, the nature of the work conducted in animal welfare projects often does not align well with established institutional ethical review committees and structures. There are multiple reasons for this. Ethical review is mandatory for most university researchers and academic institutions [5,6]. Non-governmental, not-for-profit organizations without university or government affiliations or funding are often not mandated to conduct ethical review. This describes a majority of animal welfare organizations. Moreover, even if these organizations wished to conduct such a review, they lack the institutional structures to do so.

Furthermore, institutional ethical review structures and processes tend to be designed for research involving humans or research involving animals, not for research that affects both [5,7]. (The names of bodies tasked with conducting ethical review vary by country and institution. Examples of committees established to oversee research involving animals include Institutional Animal Care and Use Committees in the United States, Animal Welfare and Ethical Review Bodies in the United Kingdom, and Animal Ethics Committees in Australia. Examples of committees established to oversee research involving humans include Institutional Review Boards, Independent Ethics Committees, Ethics Committees, and Research Ethics Committees.) Ethical review of studies involving animals is grounded in biomedical and laboratory research. Consequently, many questions, requirements, and procedures are ill suited for studies involving animals with owners or guardians, studies that take place in communities, and studies that involve non-experimental social science [5,6,7].

The biomedical and laboratory research orientation also means that decisions regarding acceptable research are typically based on a harm-benefit analysis and application of the “3Rs”: Replacement, Reduction, and Refinement [8,9]. This presumes that harms will be experienced by the target animals, and the populations (human or non-human) that benefit will differ from those in the research [7].

Much of the work conducted to advance animal welfare takes place in settings other than a laboratory. It therefore impacts not only target species and individuals, but also those who affect and are affected by them: non-target species; human guardians, owners, and community members; and the broader environment. Particularly in field contexts or initiatives involving animals with an owner or guardian, there is a need for ethical review structures to have the flexibility to include non-human animals as well as people, and to ensure that personal information is protected. There would also be value in structures that could accommodate studies designed to benefit the individual participants, or others of the same species. In such instances, the “Replacement” and “Reduction” components of the 3Rs may not be applicable, as there is—or could be—benefit to the individuals in the study. Finally, there would be value in having flexibility to accommodate research involving various levels of invasiveness and risk to the wellbeing of animal participants.

This commentary details one United States-based organization’s struggle to find ethical guidelines and support for conducting non-traditional field-based animal welfare studies, as well as our experience establishing an Ethical Review Board (ERB) to evaluate organizational initiatives. It is hoped that sharing content of the ethical review process, as well as challenges and learnings from it, will support other organizations and individuals seeking to ensure that innovation for animal welfare meets high ethical standards.

The content of this Commentary is adapted from *Ethical decision-making: Practical guidance & toolkits on ethical decision-making and considerations for field projects targeting dogs and cats* [10]. The intent was that an ERB such as that described below would be used in two specific contexts. The first context would be by nonprofit organizations or other entities for which an Institutional Review Board (IRB), Institutional Animal Care and Use Committee (IACUC), or equivalent does not exist. In other words, without the ERB, the entity would otherwise conduct the project or research without undergoing any ethical review; the ERB would therefore be a way to instill some ethical review and reflection in the process of preparing the study, project, or research. The second context would be by entities undertaking initiatives for which an IRB, IACUC, or equivalent seems unsatisfactory and unresponsive to the nature of the initiative and needs of the entity conducting it. In this circumstance, the questions might be considered in the process of preparing for the initiative, prior to submitting a proposal to the enforceable and legally required ethical review body. The ERB described here is not intended to replace an IRB, IACUC, or equivalent in instances where such an entity exists, but instead to address gaps and limitations that exist in some official institutional ethical review processes, and for certain types of studies.

## 2. Background

The mission of the Alliance for Contraception in Cats & Dogs (ACC&D) is to advance non-surgical sterilants and contraceptives for cats and dogs, and to promote their global accessibility [11]. Much of the research on new technologies is conducted in academic and medical institutions with funding from Michelson Grants in Reproductive Biology [12], an initiative of the Michelson Found Animals Foundation [13]. ACC&D works as an advocate for new technologies and an independent source of information on those that are available. The organization also spearheads projects to help ensure that, as new non-surgical fertility control options are developed, they will be implemented effectively, safely, and humanely.

In the period 2015–2016, ACC&D initiated two studies, both of which were of a new nature for the organization. One evaluated the potential of a non-surgical fertility control vaccine for free-roaming cats [14,15]; the other, a new ear marker designed to identify free-roaming dogs as having been sterilized without surgery or vaccinated against rabies [16]. The first study took place in a customized, indoor-outdoor facility designed to simulate aspects of a free-roaming cat colony while also ensuring that cats had a comfortable, enriched, safe environment and unlimited food and veterinary care. The latter took place with owned dogs in conjunction with a large-scale rabies vaccination campaign in a Kenyan Maasai pastoralist community.

ACC&D worked with academic partners who required institutional ethical review and, in both cases, had the protocol reviewed by an Institutional Animal Care & Use Committee (IACUC). However, had ACC&D conducted these studies independently, institutional ethical review would not have been mandated, despite the organization’s desire for some form of independent validation.

In many ways, the experience of ethical review for both studies was inadequate and unsatisfactory. This was especially true of the marking study, whose core ethical quandaries could not be adequately addressed given the structure of the IACUC review process. Given that the study took place in the field with owned dogs, it needed to simultaneously protect both human and animal interests and welfare. Like many animal welfare initiatives, whether or not they involved a research component, the study included problematic power differentials. The intent was for trained community members, communicating in Maasai, to obtain permission for a dog’s participation. However, the realities of a field environment meant that in some instances Kenyan veterinarians from Nairobi, speaking in Kiswahili, sought permission. Although use of pressure was prohibited—and unnecessary given owners’ overwhelming desire for their dogs to participate—the veterinarians had significantly more privilege than community members. Moreover, even when a peer sought permission, it was clear that the veterinarians and a white American were managing the study. This likely influenced some participants’ desire to volunteer their animals. Perhaps more fundamentally, the United States-based IACUC that reviewed the proposal was part of an institution with little connection to, and likely very little knowledge of, the community in which the study took place. It did not require ethics approval from a local institution or any other form of local perspective, knowledge, or expertise, for this particular study.

The IACUC process, and the Committee’s questions prior to a rapid approval, further highlighted the fact that traditional animal care and use committees are not structured well for field projects in which both animals and humans—owners, guardians, and the broader community—have a major stake. They were established to evaluate traditional laboratory research, and their design reflects this [4].

In this study, there was no way to bridge humans and non-human animals when it came to institutional ethical review, which is inevitable given that review bodies that comply with U.S. federal policies and guidelines focus on protecting either animals or humans, but not both simultaneously. These limitations to oversight and protections also hold true for many others countries’ ethical review structures. Moreover, there were few resources available providing guidance on how to conduct humane, ethical research when stakeholders span not only different countries, communities, and economic, educational, language, racial, and social backgrounds, but also different species.

## 3. New Resources for Ethical Review

In response to the “gaps” in ethical guidance identified as part of the two projects described above, ACC&D published a guide, *Ethical decision-making: Practical guidance & toolkits on ethical decision-making and considerations for field projects targeting dogs and cats* [10] (“ethics guide”). It aims to aid organizations and individuals in ensuring that ethical issues receive as much focus as the many other considerations for a successful project.

ACC&D used this guide as a foundation to establish an internal ERB, whose debut involved reviewing a small study evaluating a different marking method than was trialed in Kenya. This new marking method involved an ear tattoo applied with a microneedle patch, a technology that had previously been used to vaccinate dogs against rabies and was shown to cause less discomfort than a traditional injection [17,18]. This time, the study took place in Florida, and the veterinarian trained to apply the patches did so on his own pets. A veterinary anesthesiologist was charged with monitoring pain during and immediately following application of the microneedle tattoo; the owner (and veterinarian) performed monitoring on subsequent days, by which time pain or discomfort was not anticipated based on prior studies of a microneedle patch.

The purpose of this ERB is to supplement a mandated institutional ethical review and offer a more comprehensive ethical review process for a study that should account for the interests and needs of both people and animals, as well as unique considerations of a study that takes place in a field context. We believe that, short of mandated ethical review processes designed to protect human and non-human animals simultaneously, this is a worthwhile endeavor for entities (NGOs, government agencies, academic programs) to pursue.

As with our prior studies, ACC&D partnered with an academic institution and received IACUC approval. However, the IACUC process did not address key ethical issues for this non-traditional study, which included treatment of five pets in their home. Therefore, ACC&D supplemented with an independent review focusing on ethical questions not addressed as part of an IACUC process. The ERB review took place prior to IACUC submission. It was not intended, nor is it suggested, that the ERB described herein replace mandated institutional ethical review.

What follows is a discussion of ERB membership, materials, process, and lessons learned from one organization’s inaugural ERB review. It is shared in hopes that other organizations, or individuals whose work with animals also impacts owners, guardians, and communities, will consider the use of an ERB to better safeguard all stakeholders.

### 3.1. ERB Member Selection

ERB member selection was guided by the skill sets and personal qualities in Table 1 and Table 2. It was intentional that five of the six members have extensive animal welfare backgrounds and knowledge. The professions and backgrounds represented include veterinarians, a veterinary theriogenologist, an attorney, an animal behaviorist, scientists and researchers, and animal welfare professionals, with expertise that includes ethics and work in less-developed regions.

The Board had two meetings by conference call, each scheduled for 90 min and held two weeks apart, followed by a final solicitation for feedback via e-mail.

### 3.2. ERB Materials and Process

In advance of the first meeting, the ERB was provided with the study protocol and forms (33 pages); a copy of ACC&D’s ethics guide (160 pages), with advice on specific sections on which to focus; and 14 questions created and answered by ACC&D staff (9 pages). The questions were divided into three sections, with instructions that they be answered by the Principal Investigator or alternative project lead; that they be reviewed in conjunction with the project protocol; and that they be answered in as much detail as possible, making references to the project protocol and forms as necessary. They are listed below.

1. Study design, protocol, partners, funding
  a. What work has taken place to determine if the project is redundant, what value it is likely to add, and could it be improved?
  b. Are the project’s decision-making processes, data quality, and data management sufficiently rigorous to ensure meaningful results and learnings? Please explain.
  c. Who are the partners and personnel in the study? What was the process for selecting them?
  d. Could someone potentially view funding or partners for the project as presenting a conflict of interest? Why or why not? If a conflict of interest could be identified, how has it been addressed?
  e. Are any ethical issues outstanding in the study protocol? Please explain why or why not.
2. Animal welfare considerations
  a. What level of anticipated risk will the animals face from the project’s start to finish? How have risks been determined, and are they justified given the anticipated benefits of the project?
  b. How and why were the particular animals in the project selected? How and why were the numbers of animals in the project selected?
  c. Will the project personally benefit the animals involved? If yes, how? If no, how do you justify their participation or use in the project?
  d. What steps have been taken to ensure that the animals in the project enjoy good welfare, both physically and psychologically, from the very start to the very finish of the project?
  e. What safeguards and endpoints are in place to ensure that animals in the projects will not suffer?
3. Human stakeholders
  a. Who are all the stakeholders in the project? (If the community is impacted in any way, please delineate who/what comprises the community.)
  b. What has been done to engage each stakeholder group? What is the evidence that each stakeholder group is engaged in and supportive of the project? Are there any competing interests among stakeholders and, if so, how have they been addressed?
  c. What has been done to ensure that informed consent has been received for any human participation in the project, and informed permission for any animal participation? Were there any challenges to gaining informed consent/permission and, if so, how were they addressed?
  d. Are there any aspects of human stakeholder engagement that require consideration? If no, why not? If yes, what are they and what has been done?

These questions were an alternative to those typically found in an IACUC application in that they looked at the study holistically, with consideration of the ethical implications of the study for every being involved and desire to protect the welfare of all participants. The core issues they were intended to evaluate included:

- Potential conflicts of interest;
- Redundancy of research (not necessarily a negative quality if prior outcomes were positive) and value of research to animal welfare and/or health;
- Project quality (poor quality is arguably unethical, because potential benefits will not offset potential risks);
- Risks to animals and anticipated risk/benefit ratio to both participating animals and other members of their species;
- Anticipated physical and psychological welfare of animals throughout the study;
- Safeguards and endpoints established to prevent animal suffering;
- Identification of *all* project stakeholders, both individuals and communities;
- Stakeholder engagement, including its quality and breadth, evidence of stakeholder support, and resolution of any competing interests;
- Informed consent for any human participation in the project;
- Informed permission for participation of non-human animals based on the principles of informed consent for one’s own participation, but with recognition that animals cannot themselves consent. (The Council for International Organizations of Medical Sciences [20] addresses the nuanced distinctions between consent, assent, and permission for participation in health-related research involving humans, and we extrapolated the principles to non-human animals).

The questions asked, and the issues they raised, would be appropriate for use with the majority of studies involving human and non-human animal stakeholders. Although questions were designed for a study involving a research component, many of the issues would also be applicable for any animal welfare-focused field project with an element of innovation and, by extension, uncertainty regarding how stakeholders will respond to and benefit from the intervention.

### 3.3. The First Meeting

The first meeting was designed for the ERB to provide feedback on ethical concerns related to the proposed study following review of (extensive) advanced reading materials. ACC&D staff joined the meeting to facilitate member introductions, review goals for the ERB, and answer questions regarding the study and protocol. Staff then left the call. In the absence of staff, members were provided with the following questions to guide discussion:

1. What, if any, questions or concerns do you have regarding the study design, protocol, partners, and funding? (If applicable, do you believe that these concerns are resolvable? Do you have any recommendations for how to address?)
2. What, if any, questions or concerns do you have regarding the welfare of animals involved in the study? (If applicable, do you believe that these concerns are resolvable? Do you have any recommendations for how to address?)
3. What, if any, questions or concerns do you have regarding the welfare of human stakeholders in the study? (If applicable, do you believe that these concerns are resolvable? Do you have any recommendations for how to address?)
4. What, if any, changes would you recommend to the protocol to support proceeding with the study?

Notes were recorded and provided to staff by the ERB Secretary.

### 3.4. The Second Meeting

In advance of the second meeting, ERB members were provided with a revised study protocol and forms (41 pages) and point-by-point responses to concerns and suggestions included in the Secretary’s notes (4 pages). The meeting focused on changes made to the protocol following the first meeting and ERB members’ outstanding questions and concerns.

Similar to the first meeting, the Secretary took notes, circulated them to other ERB members for approval, and subsequently sent them to ACC&D staff. The notes listed remaining concerns and suggestions.

Because outstanding suggestions were very limited, the ERB and ACC&D staff together determined that a third call was unnecessary. ACC&D staff made tweaks to the protocol and forms and developed point-by-point responses to the ERB members’ suggestions; both documents were sent to the ERB for final approval by e-mail.

### 3.5. Ethical Review Board Feedback

ERB members’ feedback on the study spanned from predominantly pragmatic to wholly ethical considerations. There was particular focus on each animal’s behavior and relationships: whether the protocol and pain scale could capture subtle signs of pain or distress, how to avoid bias when behavior was monitored by the animals’ owner, and whether the tattoo application would have any effect on the social life of the animals—their relationships with each other and human members of the household. There was extensive discussion of welfare: the predicted effect of the microneedle tattoo on the animals’ well-being, how that effect could be evaluated, and whether the study included adequate steps to attenuate and mitigate any potential welfare infringements. There was also discussion of the ethical implications of treating one’s own animals as part of a study or project, and whether this could create a problematic precedent. It was additionally suggested that there be a retrospective ethical review upon completion for critical reflection on the project.

### 3.6. Learnings and Recommendations

The meeting yielded several learnings and areas for improvement:

#### 3.6.1. “Pragmatic” vs. Ethical Considerations

The ERB was created to focus on the ethical implications of a proposed project or study. However, in order to address ethics, it is necessary to understand pragmatic and technical considerations—something more akin to a traditional IACUC review.

This technical-focused discussion (which took place at the start of the first meeting) took longer than was anticipated, likely in part due to the length (33 pages) and complexity of the protocol being reviewed. Due to the expertise and experience of ERB members, they also offered suggestions regarding aspects of the proposed study that were not directly related to ethics. The feedback was valuable and strengthened the protocol, but it did lead, at times, to digressions from the main purpose of the meeting.

This experience reveals the need to satisfy an ERB’s need for a comprehensive understanding of the protocol before it turns to an ethics-focused discussion. There would likely need to be some trial and error and consideration of the specific project plan or protocol.

Relatedly, it reveals the need to determine how best to stay focused on ethical considerations when the qualifications of ERB members permit them to offer broader feedback and support. Members noted that thorough responses to the ethical considerations questionnaire addressed many of the questions they would have otherwise had regarding the ethical underpinnings of the study. This no doubt helped to make the process more efficient, and completing these questions, or an adaptation of them, in advance is strongly recommended. Future planning for this likely scenario could also include enforcing a focus on ethics specifically.

In light of both these needs, which are potentially at odds with one another, and depending on the complexity of the project or study, 90 min might also be inadequate for the first meeting.

#### 3.6.2. Maintaining ERB Confidentiality

It is essential to ensure that the ERB has sufficient time for confidential discussion to ensure that all feedback, including negative feedback, is captured. For this review, the time spent on technical questions at the beginning of the first meeting reduced the time available for confidential discussion. Plans for discussion of technical aspects of the study and time during which staff are present must preserve time for confidential discussion.

#### 3.6.3. ERB Leadership

For the inaugural ERB meeting, a member volunteered to serve as Secretary, but no one volunteered as Chair. As a result, ACC&D staff assumed a leadership and facilitation role during their participation in the meeting. In the course of a debrief, everyone agreed that it is essential to have an ERB Chairperson, particularly to ensure a smooth meeting without the presence of those implementing the study. It might be advisable to select ERB members who are known to be willing to serve as, at minimum, Chair and Secretary.

#### 3.6.4. Is the ERB Binding?

A “binding” ERB would mean that the ERB could state that a project cannot proceed in its proposed form—or at all. It would give the ERB authority consistent with that of conventional institutional ethical review bodies. The alternative is for the ERB to have a supervisory or guidance role, but not ultimate decision-making control.

The authority of ACC&D’s ERB was not established in advance. ACC&D staff were deferential to ERB guidance; Board members’ expertise was deliberately sought and highly valued. Although most recommendations and requests were incorporated without question, in a handful of instances, dialog between ERB members and ACC&D staff yielded resolution regarding how to proceed, meaning that the authority of the ERB to approve or reject the project was never an issue.

Such an outcome is not guaranteed and, in short, the authority of the ERB should be established in advance. In general, we would recommend that it be given authority to reject a study based on ethical grounds. However, this may not be the right decision in all instances, with possible variations based on whether the ERB is acting as a supplement or an alternative to an institutional ethical review body (e.g., IACUC, IRB, or equivalent), and the experience and expertise of the ERB members. Of note, regardless of whether the ERB’s decision is binding, the ERB would not replace an official institutional ethical review body when such review is required for the study. In ACC&D’s particular situation, the ERB conducted a review of the study and staff made changes to the protocol prior to submitting for IACUC approval. Therefore, the question of whether the ERB is binding would apply to whether the Board could require that changes be made, or could stop a study from proceeding altogether, before IACUC approval was sought.

#### 3.6.5. ERB Recommendation and Decision-Making Process

One question that arose during the inaugural ERB meeting is whether ERB decisions and recommendations are based on consensus or majority vote. Members had differences of opinion on multiple issues, including some that were pivotal to the study design. With this particular ERB, discussion of these issues was productive, and members ended up in general agreement on how to proceed. However, this is not guaranteed. Determining what to do in instances of disagreement is essential, particularly if the ERB’s recommendation is binding.

## 4. Other Ethical Review Models

A small number of entities have recently identified gaps in ethical oversight similar to those discussed in this paper and have worked to address them. One is the Brooke, a London-headquartered nongovernmental organization dedicated to improving the lives of vulnerable working horses, donkeys, and mules around the world by working with people, communities, and organizations [21]. The Brooke has established an Animal Welfare and Ethical Review Body (AWERB) [22]. Its scope is more expansive than indicated in the United Kingdom’s official AWERB materials [19], with guidelines and principles that protect both animal and human interests: animal health and welfare, informed consent and transparency, human health and well-being, intervention and cessation of the study, the “3Rs,” and environmental impact.

It is also relevant to consider ethical review of clinical research involving client owned animals that is conducted through veterinary clinics rather than academic institutions. Bertout et al. [6] address the fact that researchers conducting such studies in the United States do not necessarily have access to an IACUC, resulting in the absence of an enforceable legal requirement and limited guidance on standards for an ethical review process. Recognizing this gap in the United Kingdom, in 2019, the Royal College of Veterinary Surgeons established a permanent Ethics Review Panel for practice-based members without alternative access to ethical review of veterinary clinical trials. The research proposal application questions address ethical consideration of both animals and humans [6,23]. While there are certainly differences between companion animal welfare initiatives and clinical studies conducted by a veterinary practice, there are also ample ways in which ethical considerations might overlap. Ethical review of clinical studies in a veterinary practice could serve as a model that might improve ethical oversight of projects conducted within the animal welfare sector.

## 5. Conclusions

There is no doubt that innovation, which can easily blur with research, is important for advancing animal welfare. When institutional ethical review is not mandated, or when it is mandated but insufficient at addressing the specific ethical challenges that can arise with companion animal species and field work that impacts populations beyond the target individuals, it is incumbent upon organizations and individuals to develop strategies to ensure that their work is ethically defensible and presents minimal risks for those intended to benefit. A thoughtfully and carefully created Ethical Review Board is one such strategy.

## Figures and Tables

**Table 1:** Ethical Review Board membership requirements: Knowledge and Expertise. The content of Table 1 was developed by Dr. Lou Tasker and originally published in [10].

Knowledge/Skills	Profession	Representing Stakeholder/Stakeholder Issues
Cat/dog behavior and welfare Conduct of observational behavioral studies Multidisciplinary welfare assessments Human-animal bond/relationships Study design, data analysis	Animal welfare & behavior scientists	Animals Owners/guardians Variation in human-animal relations Scientific quality
Cat/dog health and welfare Field conditions—practical limitations to practicing veterinary medicine and surgery in the field	Veterinarians	Animals Owners/guardians Veterinary practices/veterinarians in the field
Human participants in quantitative and qualitative research in a range of field settings Human-animal bond Psychology of owners/guardians Community-related factors Study design, data analysis	Social scientists Psychologists Ethnobiologists	Human well-being Owners/guardians Communities Impact of the human-animal bond Scientific quality
Risk/hazard assessment Conduct of field trials Converting data derived from laboratory studies to characterize risk in the field Compliance	Statisticians Veterinarians with field experience	Animals Environment Understanding of risks and hazards in the field Scientific quality
Bioethical perspectives Asking important ethical questions Guiding ethical debate and deliberation	Ethicists/bioethicists	Ethics Bioethics
Wider stakeholder perspectives Local stakeholder perspectives Vulnerabilities relating to other stakeholders posed by intervention or innovation	Lay members	Wider stakeholders Openness and Transparency

**Table 2:** Ethical Review Board membership requirements: Personal Qualities. The content of Table 2 was developed by Dr. Lou Tasker and published in [10]. The qualities were originally published in and are adapted from [19].

Being open-minded, fair, and impartial Being confident to express a personal view in a non-confrontational way even if the view is considered controversial Being prepared to listen and respond to differing views and not be unnecessarily judgmental Being prepared to think outside the box and have the confidence to question the status quo Having realistic expectations of what can be achieved Having the time and commitment to make an active and informed contribution and do the role justice

## Data Availability

No new data were created or analyzed in this study. Data sharing is not applicable to this article.
